# Diabetogenic elevated childhood total fat in South Asian and Black African/Caribbean people relates to adverse early life growth and low socioeconomic position compared with White people in the UK

**DOI:** 10.1007/s00125-025-06473-9

**Published:** 2025-06-27

**Authors:** Kishan Patel, Sophie V. Eastwood, Jonathan C. Wells, Nish Chaturvedi, Charis Bridger Staatz

**Affiliations:** 1 Research Department of Population Science and Experimental Medicine, Institute of Cardiovascular Science, University College London, London, UK; 2 Centre for Longitudinal Studies, UCL Social Research Institute, University College London, London, UK; 3 UCL Great Ormond Street Institute of Child Health, London, UK

**Keywords:** Anthropometry, Birthweight, Childhood, Ethnicity, Fat-mass index, Growth, Socioeconomic status, Type 2 diabetes mellitus

## Abstract

**Aims/hypothesis:**

Excess type 2 diabetes mellitus in minority ethnic groups remains unexplained, although greater fat mass makes a strong contribution. We hypothesised that height and weight through infancy in South Asian and Black African/Caribbean subgroups is more adverse than in White populations. These, allied to poor socioeconomic position, determine greater fat mass at age 7 years.

**Methods:**

We report a secondary analysis from the UK Millennium Cohort Study, including 12,280 births of White ethnicity, and 358 of Indian, 650 of Pakistani, 268 of Bangladeshi, 163 of Black Caribbean and 277 of Black African ethnicity between 2000 and 2002. Birthweight was reported, and heights and weights were measured at ages 3, 5, 7, 11, 14 and 17 years. Bioimpedance captured fat mass, indexed to height, at ages 7, 11, 14 and 17 years. Standardised differences in anthropometry, using the White group as the comparator, were calculated. We explored the effect of early growth on ethnic differences in fat-mass index at age 7 years. Confounders included maternal anthropometry, smoking, infant breastfeeding, education, parental income and area-level socioeconomic deprivation.

**Results:**

All minority ethnic subgroups had lower birthweight and accelerated infant height and weight growth compared with White children. By age 3 years, mean height was greater in all minority ethnic groups than in White children. This height advantage was progressively lost, first in Bangladeshi children. By age 17 years in boys/girls, Indians were 1.77/2.48 cm, Pakistanis 2.24/3.44 cm, Bangladeshis 4.83/5.95 cm and Black Caribbeans 1.64/0.49 cm shorter than White children. Heights were equivalent in Black African children. By age 17 years, all South Asian children were lighter, and Black African/Caribbean children heavier, than White children. The anthropometric gradient by ethnicity in children mirrored that in mothers. Girls from minority ethnic groups were more likely to be menstruating by age 11 years than White girls (range 12–27% vs 9%). At age 7 years, standardised fat-mass index (kg/m^2^) in boys/girls was 0.17/0.01 SDs greater in Indian, 0.21/0.04 in Pakistani, 0.18/0.16 in Bangladeshi, 0.48/0.35 in Black Caribbean and 0.37/0.75 in Black African children than in White children. These differences persisted to age 17 years. Weight gain to age 3 years, and in Black Africans/Caribbeans, adverse individual and neighbourhood socioeconomic position, contributed to ethnic differences in fat mass.

**Conclusions/interpretation:**

Minority ethnic groups in the UK have poorer childhood growth than White children, achieving shorter height, greater fat mass and earlier female puberty. Mirroring of maternal and offspring ethnic subgroup gradients in height and weight indicates intergenerational transmission. Persistent adverse socioeconomic circumstances perpetuate ethnic adversity in early life accrual of body fat.

**Data availability:**

All MCS data used in this analysis are available from UK Data Service with an end user licence (https://ukdataservice.ac.uk/find-data/).

**Graphical Abstract:**

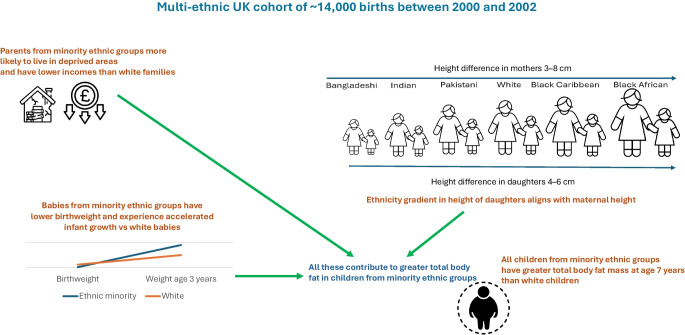

**Supplementary Information:**

The online version of this article (10.1007/s00125-025-06473-9) contains peer-reviewed but unedited supplementary material.


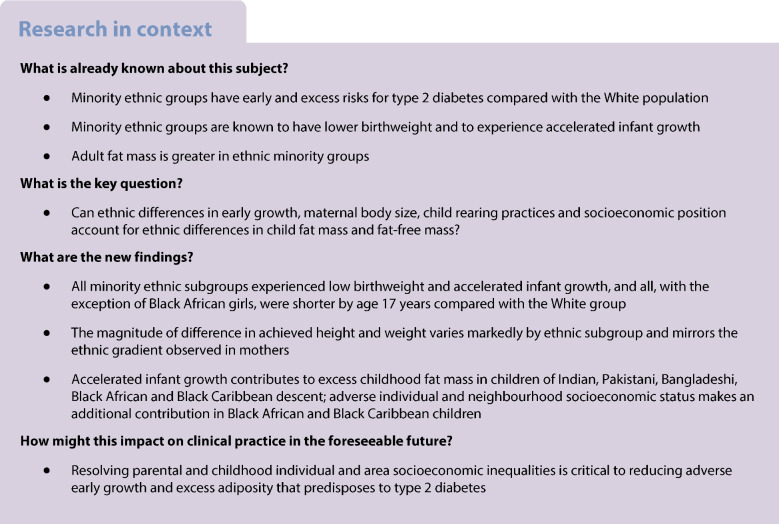


## Introduction

People of South Asian and of African (Black African and Black Caribbean) ethnicity have markedly greater risks and earlier age of onset of type 2 diabetes mellitus than White Europeans [1, 2]. Explanations for this premature excess are not entirely understood, although greater total body fat and adverse fat distribution are major contributors [2]. Poor in utero development, proxied by low birthweight, coupled with adverse patterns of accelerated early postnatal growth are associated both with elevated total body fat and with type 2 diabetes-associated traits at birth and in childhood, and likely make a strong contribution to premature disease onset [3, 4]. Low birthweight and accelerated early height growth have been reported overall in both South Asians and African Caribbeans [5] but not by ethnic subgroup and not in association with fat mass (FM). Separately, while low fat-free mass (FFM) is also evident in South Asians [6], making an additional contribution to type 2 diabetes risk [7], lean mass appears greater in people of African descent [6]. These previous studies did not have historical growth data so associations between growth and FM could not be explored. Additionally, central fat deposition, viewed as a hallmark of adverse adiposity patterns in South Asians and also contributing to excess type 2 diabetes, is not always consistently observed [1] and does not appear to feature in people of African descent [2].

While biology has been invoked to account for ancestral differences in body habitus and type 2 diabetes risk, socioeconomic factors also strongly influence differential growth and fat distribution patterns. Socioeconomic status (SES) has had a marked impact on secular trends in height and weight [8], and both individual and neighbourhood measures of socioeconomic position determine divergent fat-mass trajectories [9]. The more adverse SES of minority ethnic groups in the UK strongly contributes to lower birthweights compared with the White population [10]. Both ethnicity and SES have also been associated with earlier age at menarche, itself associated with adiposity [11].

We hypothesised that early life growth patterns in the UK differ by ethnic subgroup. People of South Asian and African ethnicity will be shorter and heavier by late adolescence compared with White people, and pubertal maturation will occur earlier. While both ancestries will have greater FM than White people, FFM will be lower in South Asians but higher in children of Black Caribbean and Black African descent. Ethnic subgroups, by parental country of origin, will have different growth patterns in association with differences in SES. These different growth patterns, coupled with adverse socioeconomic circumstances, will account for ethnic differences in both FM and FFM in childhood.

## Methods

These are secondary analyses of the Millennium Cohort Study (MCS). The MCS is a UK nationally representative birth cohort, oversampling regions with higher concentrations of minority ethnic populations. It consists of 19,244 families who had children born in the UK between the years 2000 and 2002. Recruitment was at 9 months of age of the child for 18,552 families, and 3 years of age for 692 families. Questionnaires to parents captured information around birth and were repeated when children were aged 3, 5, 7 and 11 years. At age 14 years, both the child and parents provided information, and at age 17 years, the young people responded themselves. Ethics approval was obtained by the National Health Service Research Ethics Committee up to age of 14 years, and the National Research Ethics Service at age 17 years. Informed parental consent was obtained in advance of data collection up to age 14 years. At age 17 years, informed verbal consent was obtained by the participant. Participants were able to refuse completing any element of the study or withdraw at any time, even at younger ages when written consent was only obtained from the parent.

Parents were asked to assign ethnicity of their children using the UK census groupings of White, Indian, Pakistani, Bangladeshi (comprising South Asian ancestry), Black Caribbean and Black African [12]. Specific mixed-ethnicity groups (e.g. Black Caribbean/White, Pakistani/White) or other ethnicities were small and therefore excluded from this analysis. Sex of child and parents were self reported by the respondent, usually the mother, at recruitment.

The main outcome variables were height from age 3 to age 17 years, weight including birthweight to age 17 years, and fat mass index (FMI) and fat-free mass index (FFMI) at age 7, 11, 14 and 17 years. Anthropometric measures have been described [9]. Briefly, body fat percentage (FM%) was collected by foot-to-foot bioelectrical impedance analysis (BIA) using the Tanita (Bf-522W; Tokyo, Japan) scales, performed by trained interviewers following standardised protocols. Equations used to calculate FM% were those used by the manufacturer. Height and weight were measured before BIA assessment and were inputted into the scales to enable output of FM%. FM was calculated by dividing FM% by 100 and multiplying by total body weight (FM = [FM%/100] × weight). FMI was derived by indexing FM to height squared (FMI = FM/height^2^). FFM was calculated by subtracting FM from total body weight (FFM = weight−FM), and FFMI was obtained by indexing FFM to height squared (FFMI = FFM/height^2^). The FMI/FFMI ratio at age 7 years was calculated. In girls, onset of menstruation by the time of the questionnaire at age 11 years was used as a proxy for puberty, as previously reported [13].

Factors accounting for differences between ethnicities in FMI and FFMI at age 7 years included the growth measures of birthweight, relative weight gain from birth to age 3 years (calculated by subtracting birthweight from weight at age 3 years, and dividing by birthweight in kg; this captures the early postnatal accelerated growth period), waist circumference at ages 5 and 7 years, and waist/height ratio at ages 5 and 7 years. Maternal factors included self-reported maternal height and pre-pregnancy weight, and calculated BMI, smoking status and breastfeeding duration to 4 months captured at recruitment. SES was assessed using the Index of Multiple Deprivation (IMD), an area measure of deprivation at birth, as well as individual measures including parental education (college degree/no college degree) and highest parental income quintiles at birth of the study participant. Ethics approval for data collection was granted by the NHS Research Ethics Committee up to age 14 years, and then by the National Research Ethics Service at age 17 years.

### Statistical analyses

The analytic sample comprised all cohort members with complete information on ethnicity and at least one response for each outcome and factor included in multivariable models from birth to age 17 years. Sample design weights were used to correct for MCS cases having unequal probabilities of selection that result from the stratified cluster sample design. The data were survey-weighted, with MCS sampling weights applied to each statistical model to ensure the results accurately represent the UK population [14].

Multiple imputation was performed to address missing data on factors included in multivariable models, under a missing-at-random assumption. Twenty imputations were generated, accounting for item non-response and dropout. Birthweight-related variables and ethnicity were included in the imputation model as predictors of missingness [15]. This approach ensured that variability and uncertainty associated with the missing data were properly accounted for, leading to more robust and reliable statistical analyses, preserving variable relationships and minimising bias.

Descriptive analyses calculated averages (mean or median, depending on whether the variable was normally distributed or not) and percentages of maternal characteristics, parental sociodemographic indices and early life anthropometric measures, stratified by ethnicity and sex. Standardised measures of height, weight, FM and FFM were calculated at each age using the SD of the White group.

Regression analyses calculated coefficients for FMI and FFMI, comparing ancestral groups with the White group at age 7 years (the first time these were measured), stratified by sex. Three models were employed: Model 1, unadjusted; Model 2, adjusted for early life growth (i.e. weight gain from birth to age 3 years); and Model 3, fully adjusted for maternal, early life and SES factors.

All analyses were performed using STATA version 18 (StataCorp, www.stata.com). Graphs were generated using R version 4.4.1 (R Foundation for Statistical Computing, www.R-project.org/.)

## Results

### Demographic characteristics of the sample by ethnicity

Of the 18,786 mothers of participants who had any interaction with the ethnicity module at recruitment, 48 did not complete it. A further 562 were excluded as being of ‘mixed ethnicity’, and 308 as being ‘other ethnicity’. The remaining 17,868 recruits’ ethnicity was as follows: White, 15,491; Indian, 470; Pakistani, 903; Bangladeshi, 368; Black Caribbean, 247; and Black African, 389. From there, exclusions were only on the basis of not having at least one data point for our outcomes. The ethnicity of those remaining was: White, 12,280; Indian, 358; Pakistani, 650; Bangladeshi, 268; Black Caribbean, 163; and Black African 277 (Table 1).

**Table 1:** Maternal characteristics, parental sociodemographic indices and offspring birthweight, early life weight gain, waist-to-height ratio, FMI/FFMI ratio and, in girls, early puberty, by ethnicity

Characteristic	White *n*=12,280	Indian *n*=358	Pakistani *n*=650	Bangladeshi *n*=268	Black Caribbean *n*=163	Black African *n*=277
Maternal height (m)	1.64±0.08	1.60±0.08	1.61±0.08	1.57±0.09	1.65±0.08	1.65±0.10
Maternal weight (kg)	64.2±15.0	56.8±13.4	60.8±14.7	58.5±15.6	67.9±16.9	68.3±18.5
Maternal BMI (kg/m^2^)	23.7±5.3	22.3±5.1	23.5±5.9	23.7±6.4	25.0±5.7	25.1±6.3
Maternal smoking, %	12.5	4.1	3.6	2.3	9.6	1.8
Breastfed to 4 months, %	22.6	25.6	14.5	19.2	39.5	39.8
Area deprivation (IMD)	5.8±3.2	5.0±3.9	2.7±3.7	2.5±3.7	3.4±2.8	3.0±3.2
Degree-level education, %	29.7	37.8	14.2	12.3	30.4	40.3
Highest parental income quintile at birth, %	18.8	17.8	3.7	2.2	9.8	9.0
Boys, *n*	6274	184	330	129	85	147
Birthweight (kg)	3.46±0.69	3.03±0.92	3.15±0.66	3.13±0.59	3.27±0.90	3.37±0.77
Relative weight gain, birth to age 3 years	4.57±1.37	5.14±2.56	4.87±1.30	4.93±1.38	5.20±2.40	4.98±1.09
Waist circumference (cm) at age 5 years	54.01±4.50	53.17±7.03	52.58±6.03	53.52±6.22	55.26±6.66	53.98±5.57
Waist-to-height ratio at age 5 years	0.49±0.04	0.47±0.06	0.47±0.05	0.48±0.05	0.49±0.06	0.47±0.04
Waist circumference (cm) at age 7 years	57.13±6.18	57.31±8.27	57.10±8.89	57.04±7.80	58.75±9.16	58.01±7.40
Waist-to-height ratio at age 7 years	0.46±0.04	0.46±0.06	0.46±0.06	0.46±0.05	0.47±0.07	0.45±0.04
FMI/FFMI ratio at age 7 years	0.25±0.09	0.28±0.12	0.28±0.14	0.27±0.10	0.29±0.15	0.29±0.10
Girls, *n*	6006	171	320	139	78	130
Birthweight (kg)	3.34±0.66	2.96±0.81	3.08±0.67	2.97±0.95	3.15±0.72	3.16±0.72
Relative weight gain, birth to age 3 years	4.52±1.37	5.01±2.22	4.94±1.71	5.29±3.56	5.41±3.64	5.36±1.63
Waist circumference (cm) at age 5 years	53.78±4.80	52.75±5.67	53.11±5.82	53.68±7.62	54.88±5.13	55.72±6.35
Waist-to-height ratio at age 5 years	0.49±0.04	0.47±0.05	0.48±0.05	0.49±0.07	0.49±0.04	0.49±0.05
Waist circumference (cm) at age 7 years	57.03±6.55	56.51±7.30	57.07±7.92	56.95±8.92	58.49±7.07	61.31±9.70
Waist-to-height ratio at age 7 years	0.46±0.05	0.46±0.05	0.46±0.06	0.46±0.06	0.46±0.05	0.48±0.06
FMI/FFMI ratio at age 7 years	0.29±0.11	0.30±0.11	0.30±0.12	0.31±0.14	0.32±0.12	0.37±0.16
Menstruation onset before age 11 years, %	9.0	26.9	11.8	27.2	17.9	22.8

Data are shown as mean ± SD, or %

Electronic supplementary material (ESM) Table 1 shows missing data, ranging from 1 for IMD, to 6118 for FM at age 17 years. South Asian mothers (of Indian, Pakistani or Bangladeshi descent) were shorter and lighter than their White counterparts, whereas Black Caribbean and Black African mothers were of similar height but heavier (Table 1). Breastfeeding for at least 4 months was highest among Black Caribbean and Black African mothers. All minority ethnic group mothers were less likely to smoke than White European mothers. Indian, Black Caribbean and Black African mothers were equally or more likely to be educated to degree level compared with White mothers. In contrast, all minority groups were less likely to be in the highest quintile of parental income and more likely to live in a socioeconomically deprived neighbourhood than their White counterparts. For the latter, Bangladeshi families were most disadvantaged, followed by Pakistani, Black African, Black Caribbean and Indian families.

### Weight at birth through to age 17 years by ethnicity

**Fig. 1:**
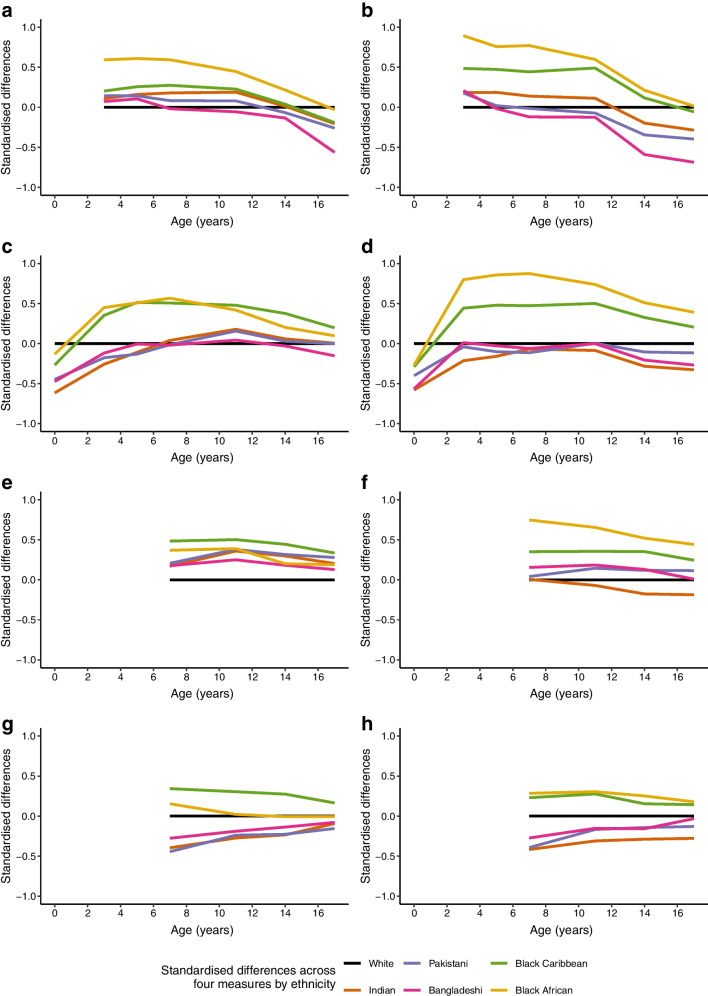
Standardised ethnic differences in height (**a**, boys; **b**, girls), weight (**c**, boys; **d**, girls), FMI (**e**, boys; **f**, girls) and FFMI (**g**, boys; **h**, girls) compared with the White group stratified by sex

Boys and girls from all minority groups had lower birthweights than White boys and girls, with the most marked differences observed in Indian children (standardised difference: −0.62 for boys, −0.58 for girls), and least in Black African children (−0.13 for boys, −0.27 for girls) (Table 1, Fig. 1 and ESM Table 2a, b). Relative weight gain to age 3 years was greater in all minority groups than in the White group (Table 1). Black Caribbean and Black African children were heavier at age 3 years, and remained so to age 17 years, compared with White children; in contrast, South Asian children were lighter than White children at all ages, except briefly for boys at age 11 years (Fig. 1 and ESM Table 2a, 2b).

### Height from infancy through to age 17 years by ethnicity

Height patterns were different. All boys and girls from minority ethnic groups were taller than White children at age 3 years (Fig. 1 and ESM Table 2a, b). However, this height advantage was progressively lost in South Asian children: by age 7 years, Bangladeshi boys and girls were shorter than White children; by age 11 years, Pakistani children were shorter than White children; and by age 14 years, Indian children were shorter than White children. In boys and girls, respectively, by age 17 years, Indian children were 1.77/2.48 cm, Pakistani children 2.24/3.44 cm and Bangladeshi children 4.83/5.95 cm shorter than White children (ESM Table 2a). Black Caribbean and Black African children also displayed a progressive loss of height advantage with age, falling to similar or lower heights than White children only by age 17 years (in boys and girls, 1.64/0.49 cm shorter in Black Caribbean children, 0.27 shorter in Black African boys, and only 0.1 cm taller in Black African girls compared with White children). The height gradient by ethnicity in girls aged 17 years reflected that observed in their mothers.

### Waist circumference by ethnicity

There were no clear differences in waist circumference and waist-to-height ratios by ethnicity at ages 5 and 7 years (Table 1).

### Ethnic differences in menstruation onset by age 11 years

Girls from minority ethnic groups were 1.3–3 times more likely to have commenced menstruation by age 11 years than White girls (Table 1).

### Ethnic differences in FMI and FFMI in childhood and adolescence

At age 7 years, standardised FMI (kg/m^2^) in boys/girls was higher in all minority groups save Indian girls (0.17/0.01 SDs greater in Indian, 0.21/0.04 in Pakistani, 0.18/0.16 in Bangladeshi, 0.48/0.35 in Black Caribbean and 0.37/0.75 in Black African children than in White children). These differences persisted through ages 11, 14 and 17 years. (Fig. 1 and ESM Tables 2a, b). FFMI was lower in all South Asian groups for both boys and girls, and lower in Black African boys than their White counterparts after age 7 years. Black Caribbean boys and girls had higher FFMI than White children at all ages. Ethnic differences in FFMI were most marked at age 7 years, with a progressive convergence to levels in White children by age 17 years (Fig. 1). The FMI/FFMI ratio at age 7 years was elevated in all minority ethnic groups for both boys and girls compared with White children (Table 1).

Adjustment for relative weight change from birth to age 3 years markedly attenuated ethnic differences in FMI at age 7 years for both boys and girls (Table 2). Multivariable models indicated an additional contribution from maternal and socioeconomic factors for Black Caribbean and Black African children, but not South Asian children (Table 2 and ESM Table 3a, b). Similar, though less marked, patterns of attenuation of ethnic differences compared with White children were observed for FFMI (Table 2 and ESM Tables 4a, b).

**Table 2:** Accounting for ethnic differences (using White group as comparator) in FMI and FFMI at age 7 years stratified by sex

Index	Boys					Girls				
	Indian	Pakistani	Bangladeshi	Black Caribbean	Black African	Indian	Pakistani	Bangladeshi	Black Caribbean	Black African
FMI										
Model 1^a^	0.24 (−0.04, 0.53)	0.29* (0.06, 0.53)	0.25 (−0.02, 0.52)	0.68* (0.16, 1.21)	0.52*** (0.27, 0.78)	0.01 (−0.24, 0.26)	0.07 (−0.13, 0.27)	0.26 (−0.11, 0.62)	0.59** (0.16, 1.01)	1.24*** (0.81, 1.68)
Model 2^b^	0.14 (−0.14, 0.43)	0.24* (0.01, 0.47)	0.19 (−0.08, 0.46)	0.58* (0.07, 1.08)	0.45*** (0.20, 0.70)	−0.09 (−0.36, 0.18)	−0.02 (−0.22, 0.18)	0.10 (−0.27, 0.46)	0.40 (−0.02, 0.82)	1.06*** (0.65, 1.48)
Model 3^c^	0.34* (0.08, 0.60)	0.27* (0.05, 0.49)	0.17 (−0.08, 0.43)	0.46 (−0.01, 0.94)	0.31** (0.08, 0.54)	0.20 (−0.07, 0.48)	−0.02 (−0.21, 0.17)	0.02 (−0.33, 0.36)	0.09 (−0.34, 0.51)	0.72*** (0.33, 1.11)
FFMI										
Model 1^a^	−0.47*** (−0.71, −0.23)	−0.53*** (−0.70, −0.36)	−0.33* (−0.60, −0.05)	0.41* (0.09, 0.73)	0.18 (−0.07, 0.44)	−0.48*** (−0.68, −0.28)	−0.45*** (−0.59, −0.31)	−0.31* (−0.56, −0.06)	0.26* (0.01, 0.52)	0.33** (0.08, 0.57)
Model 2^b^	−0.51*** (−0.75, −0.28)	−0.55*** (−0.72, −0.38)	−0.36* (−0.63, −0.08)	0.36* (0.04, 0.68)	0.15 (−0.10, 0.40)	−0.52*** (−0.72, −0.31)	−0.49*** (−0.62, −0.35)	−0.38** (−0.63, −0.12)	0.19 (−0.06, 0.44)	0.26* (0.02, 0.49)
Model 3^c^	−0.29** (−0.51, −0.08)	−0.44*** (−0.60, −0.28)	−0.27 (−0.55, 0.01)	0.25 (−0.04, 0.54)	0.03 (−0.19, 0.26)	−0.32** (−0.53, −0.10)	−0.45*** (−0.58, −0.31)	−0.40** (−0.65, −0.15)	0.00*** (1.00, −0.25)	0.05 (0.65, −0.17)

Data are shown as coefficient (95% CI)

^a^Model 1, unadjusted

^b^Model 2, adjusted for weight gain from birth to age 3 years

^c^Model 3, adjusted for maternal (height, pre-pregnancy weight, BMI, smoking, breastfeeding to 4 months), birthweight, weight gain from birth to age 3 and socioeconomic factors (IMD, parental education, parental income)

^*^p<0.05, **p<0.01 and ***p<0.001 vs White group

## Discussion

Young adults of South Asian, Black African and Black Caribbean ethnicity born in the UK at the turn of this century have distinct early life growth patterns compared with the White population. They share low birthweight and early accelerated growth, accompanied by premature growth deceleration. The magnitude of the former and timing of the latter, however, differs by ethnicity, impacting final body habitus. At age 17 years, South Asian people were lighter and shorter, while Black African and Black Caribbean people were heavier and of similar or marginally lower height compared with their White counterparts. Minority ethnic groups had greater total FM than White people but South Asian children had lower, and Black Caribbean children and Black African girls higher, indices of FFM than White children. However, the ratio of FM to FFM was uniformly greater in all minority groups at age 7 years than in White children. Low birthweight followed by greater postnatal growth in all minority groups studied here contributes to excess FMI compared with White children, with further contributions from adverse individual and neighbourhood socioeconomic position in people of Black African and Black Caribbean ethnicity. All analyses were stratified by sex, but largely, ethnic differences in anthropometry and associations with determinants were similar in both sexes.

Low birthweight in children of South Asian, Black Caribbean and Black African ethnicity in the UK is known [16], and previous analyses of this cohort concluded that this was largely due to adverse socioeconomic circumstances [10]. While accelerated postnatal growth is also recognised [17], the current analysis is the first to identify important differences in the effects this has on achieved height and weight beyond the age 5 years for different ethnicities. Relative weight gain from birth in all ethnic minority groups is of similar magnitude, and greater than that observed in White children. However, as children of Black African and Black Caribbean ethnicity have higher birthweights than South Asian children, this accelerated weight gain results in heavier children by age 3 years compared with White children, an advantage that persists through to late adolescence. Absolute weight gain in South Asian children is lower, and weight only briefly achieves levels similar to or greater than those in White boys around age 11 years, otherwise remaining lower than in White boys and girls throughout early life.

In contrast to weight, all minority ethnic groups are taller by the age of 3 years. This height advantage is also progressively and differentially lost. By age 17 years, Black African children are of similar height to, and Black Caribbean children somewhat shorter than, White children. The height advantage at age 3 years in South Asian groups is progressively lost, first in those of Bangladeshi, then of Pakistani and finally of Indian origin. By the age of 14 years all South Asian ethnicities are shorter than White children. This ethnic difference is particularly marked for Bangladeshi children, who by age 17 years are ~5.0–6.0 cm shorter than their White counterparts.

Interestingly the ethnic subgroup gradient in achieved height and weight by age 17 years in the current generation is identical to that observed in their parents. Persistence of low birthweight has been observed some generations after migration from Surinam to the Netherlands [18], and in African-Americans in the USA [19] resident in the USA for many generations. Short maternal stature and lower lean mass correlate with smaller organ mass and smaller birth canal dimensions [20, 21], which may then constrain fetal growth through both genetic and plastic mechanisms [22]. More broadly, poor in utero development as a consequence of constrained early growth of the mother, and continuing adverse maternal circumstances, may determine persistence of adverse growth patterns in migrant populations [20–22]. It should be noted that height is strongly influenced by environmental factors (e.g. in just a few decades, South Koreans have become on average 8 cm taller than their North Korean counterparts).

Continued low socioeconomic position in minority groups is, however, a unifying feature in high-income countries and others have shown that low socioeconomic position is a key determinant of these low birthweights [10]. Most previous studies focus on the period of early weight gain [23] but we show that while relative weight gain is similar across minority groups (and uniformly greater than in the White group) the absolute consequences for height and weight, allied to differential ‘wearing off’ of this rapid growth, is markedly different. Accelerated postnatal growth was observed in all minority groups, even though certain standard risk factors, such as maternal smoking, breastfeeding and maternal educational status, were often more favourable.

All minority groups displayed earlier growth deceleration compared with White children, and all minority girls were markedly more likely to experience early puberty, defined here as menstruation onset before age 11 years, than White girls. This confirms previous analyses on this cohort but notably the percentage of Black Caribbean girls, at ~18%, is far higher than previously reported (13.0%) [13], as we excluded those of mixed ethnicity from our analysis. This earlier onset of menarche will make an additional contribution to adult obesity and type 2 diabetes [24].

We confirm FMI at age 7 years is greater in all minority groups [25], and remains so through to age 17 years apart from in Indian girls. We also confirm that FFMI is lower in South Asian children but higher in children of Black Caribbean, and girls of Black African, ethnicity [6]. However, in resolution of this paradox in FFM, we also show that the ratio of FM to FFM is uniformly greater in all minority ethnic groups studied here compared with White children at age 7 years. Skeletal muscle, proxied here by FFM, is an important determinant of type 2 diabetes risk [26]. Mechanisms include impaired insulin-stimulated glucose uptake, fatty deposition, imbalanced protein synthesis and poor mitochondrial function. While bioelectrical impendence analysis captures quantity, and we show that relative quantity is lower in minority groups than in the White group, this tells us little about relative quality. Given the more adverse early life patterns of growth in minority groups, we suggest that lean mass quality may also be poorer [27] and contribute to the observed greater risk and earlier onset of type 2 diabetes.

Biological, specifically genetic explanations have been invoked to account for ethnic differences in body composition [28]. However, self-reported ethnicity is not a biological construct [29] and the importance of current lifestyle and of socioeconomic position should not be overlooked. Generational increases in height [8], by some 6 cm over 50 years in the UK, have been ascribed to better diets and to a reduction in catastrophic childhood infection. Socioeconomic status, as a determinant of access to adequate and healthy nutrition, may account for the reversal in the socioeconomic gradient in obesity [8] and for the more adverse fat patterning observed in latter generations.

Ethnic differences in FMI were most strongly associated with relative weight gain between birth and age 3 years. Birthweight and maternal pre-pregnancy weight also made independent contributions to ethnic differences in FMI. It should be noted that South Asian mothers were both lighter and shorter than their White counterparts, accounting for the multivariable estimated ethnic difference in FMI in South Asian vs White children being of greater magnitude than the unadjusted estimate, as generally lower maternal height and weight should each associate with lower rather than higher FMI in the offspring. While we distinguish maternal/early life and socioeconomic determinants of ethnic differences in FM, it should be noted that the former, such as maternal height, smoking status and offspring birthweight, are also dependent on socioeconomic factors. Thus it is likely that we have underestimated the role of socioeconomic position in accounting for ethnic differences. Additionally, similar to previous analyses of socioeconomic differentials [9], we show that individual and neighbourhood socioeconomic position each contribute to ethnic differences in FM, indicating that beyond familial circumstances, the more obesogenic environments of neighbourhood deprivation (greater density of fast-food outlets, fewer opportunities for recreation), play a role in adverse fat accumulation. Yet adverse neighbourhood socioeconomic factors, which should similarly affect people of both South Asian and of African ethnicity, cannot explain the markedly lower levels of FFM in South Asian groups. Pomeroy and colleagues demonstrated a marked reduction in stature in people from the Indian subcontinent allied to adoption of an agricultural lifestyle some 11,000 years ago, and of a low lean mass for given body size [30]. They hypothesised that these expressions of body habitus are due to historical climatic adaptation and to cumulative ecological pressures.

The MCS is representative of the UK population at birth, and efforts were made to ensure appropriate representation of minority ethnic groups. Sample attrition and attendant data missingness have been overcome by multiple imputation. We largely report absolute (standardised) differences in body habitus at each age, rather than model growth trajectories. This is a critical initial first step to identify important differences and critical time points before embarking on methods to model growth trajectory heterogeneity; such models often make a priori assumptions, require careful specification and ideally multiple approaches to compare consistency of outcomes and thus enable robustness of conclusions [31]. BIA estimates FM% and can be used to derive FM and FFM, and the respective indices. These equations make assumptions about relationships between body habitus and FM, and about the relationship of body proportions with FFM. Previous BIA validation studies against gold standard deuterium methods in UK multi-ethnic childhood and adolescent populations variously report both over- and underestimation of FM and FFM by bioimpedance in minority ethnic groups [6, 32]. Bioimpedance equations that overcome these biases have been proposed. Unfortunately, the MCS did not record direct bioimpedance measures, so that more ethnically valid derived equations could not be applied. However, as the direction of ethnic difference in FMI and FFMI remains consistent in validation studies, even though the magnitude may differ [6], we do not view this as a major limitation.

In conclusion, we demonstrate marked ethnic subgroup differences in early development that relate to achieved height and weight, body fat, FFM and reproductive maturity in the UK. Minority ethnic groups in the UK display more adverse patterns in body habitus than White children, likely determining early onset of type 2 diabetes and other chronic conditions, though further follow-up of the MCS is needed to be certain of this. The role of ethnic differences in quality, as opposed to quantity, of fat and of lean mass in accounting for the differential risk of type 2 diabetes is not known, nor is the impact of disordered early growth on tissue quality understood. Part of these ethnic differences can be accounted for by adverse individual and neighbourhood socioeconomic position, which persists across generations [33]. Addressing socioeconomic adversity is imperative if we are to resolve the intergenerational cycle of adverse growth and premature onset of chronic disease.

## Supplementary Information

Below is the link to the electronic supplementary material.

ESM Tables (PDF 269 KB )

## Data Availability

All MCS data used in this analysis are available from UK Data Service with an end user licence (https://ukdataservice.ac.uk/find-data/).

## Abbreviations

BIA: Bioelectrical impedance analysis
FM: Fat mass
FM%: Body fat percentage
FMI: Fat-mass index
FFM: Fat-free mass
FFMI: Fat-free mass index
IMD: Index of Multiple Deprivation
MCS: Millennium Cohort Study
SES: Socioeconomic status

## References

[1] Narayan KMV, Kanaya AM (2020) Why are South Asians prone to type 2 diabetes? A hypothesis based on underexplored pathways. Diabetologia 63(6):1103–1109. 10.1007/s00125-020-05132-5 32236731 10.1007/s00125-020-05132-5 PMC7531132

[2] Tillin T, Hughes AD, Godsland IF et al (2013) Insulin resistance and truncal obesity as important determinants of the greater incidence of diabetes in Indian Asians and African Caribbeans compared with Europeans: the Southall And Brent REvisited (SABRE) cohort. Diabetes Care 36(2):383–393. 10.2337/dc12-0544 22966089 10.2337/dc12-0544 PMC3554271

[3] Wells JC, Chomtho S, Fewtrell MS (2007) Programming of body composition by early growth and nutrition. Proc Nutr Soc 66(3):423–434. 10.1017/S0029665107005691 17637095 10.1017/S0029665107005691

[4] Eriksson JG, Forsen T, Tuomilehto J, Osmond C, Barker DJ (2003) Early adiposity rebound in childhood and risk of type 2 diabetes in adult life. Diabetologia 46(2):190–194. 10.1007/s00125-002-1012-5 12627317 10.1007/s00125-002-1012-5

[5] Lowe J (2016) Early adiposity rebound, birthweight, and ethnicity: evidence from the millennium cohort study. Centre for Longitudinal Studies Working Paper. London: Institute of Education

[6] Haroun D, Taylor SJ, Viner RM et al (2010) Validation of bioelectrical impedance analysis in adolescents across different ethnic groups. Obesity (Silver Spring) 18(6):1252–1259. 10.1038/oby.2009.344 19875994 10.1038/oby.2009.344

[7] Son JW, Lee SS, Kim SR et al (2017) Low muscle mass and risk of type 2 diabetes in middle-aged and older adults: findings from the KoGES. Diabetologia 60(5):865–872. 10.1007/s00125-016-4196-9 28102434 10.1007/s00125-016-4196-9

[8] Bann D, Johnson W, Li L, Kuh D, Hardy R (2018) Socioeconomic inequalities in childhood and adolescent body-mass index, weight, and height from 1953 to 2015: an analysis of four longitudinal, observational, British birth cohort studies. Lancet Public Health 3(4):e194–e203. 10.1016/S2468-2667(18)30045-8 29571937 10.1016/S2468-2667(18)30045-8 PMC5887082

[9] Staatz CB, Kelly Y, Lacey RE, Hardy R (2021) Area-level and family-level socioeconomic position and body composition trajectories: longitudinal analysis of the UK Millennium Cohort Study. Lancet Public Health 6(8):e598–e607. 10.1016/S2468-2667(21)00134-1 34332672 10.1016/S2468-2667(21)00134-1 PMC8342403

[10] Kelly Y, Panico L, Bartley M, Marmot M, Nazroo J, Sacker A (2009) Why does birthweight vary among ethnic groups in the UK? Findings from the Millennium Cohort Study. J Public Health (Oxf) 31(1):131–137. 10.1093/pubmed/fdn057 18647751 10.1093/pubmed/fdn057

[11] Morris DH, Jones ME, Schoemaker MJ, Ashworth A, Swerdlow AJ (2010) Determinants of age at menarche in the UK: analyses from the Breakthrough Generations Study. Br J Cancer 103(11):1760–1764. 10.1038/sj.bjc.6605978 21045834 10.1038/sj.bjc.6605978 PMC2994234

[12] Office for National Statistics (2023) Ethnic group variable: Census 2021. Definition of ethnic group, categories, and changes since the 2011 Census for use with research and analysis using Census 2021 data. Available from https://www.ons.gov.uk/census/census2021dictionary/variablesbytopic/ethnicgroupnationalidentitylanguageandreligionvariablescensus2021/ethnicgroup. Accessed 12 Dec 2024

[13] Kelly Y, Zilanawala A, Sacker A, Hiatt R, Viner R (2017) Early puberty in 11-year-old girls: Millennium Cohort Study findings. Arch Dis Child 102(3):232–237. 10.1136/archdischild-2016-310475 27672135 10.1136/archdischild-2016-310475 PMC5339561

[14] Center for Longitudinal Studies (2020) Millennium Cohort Study user guide (Surveys 1-5). August 2020. Available from https://doc.ukdataservice.ac.uk/doc/5795/mrdoc/pdf/mcs1-5_user_guide_ed9_2020-08-07.pdf. Accessed 27 Nov 2024

[15] von Hippel PT (2020) How many imputations do you need? A two-stage calculation using a quadratic rule. Sociol Methods Res 49(3):699–718. 10.1177/0049124117747303 39211325 10.1177/0049124117747303 PMC11361408

[16] Harding S, Rosato MG, Cruickshank JK (2004) Lack of change in birthweights of infants by generational status among Indian, Pakistani, Bangladeshi, Black Caribbean, and Black African mothers in a British cohort study. Int J Epidemiol 33(6):1279–1285. 10.1093/ije/dyh186 15155695 10.1093/ije/dyh186

[17] Sacker A, Kelly YJ (2012) Ethnic differences in growth in early childhood: an investigation of two potential mechanisms. Eur J Public Health 22(2):197–203. 10.1093/eurpub/ckr032 21441556 10.1093/eurpub/ckr032

[18] de Wilde JA, van Buuren S, Middelkoop BJ (2013) Trends in birth weight and the prevalence of low birth weight and small-for-gestational-age in Surinamese South Asian babies since 1974: cross-sectional study of three birth cohorts. BMC Public Health 13:931. 10.1186/1471-2458-13-931 24098977 10.1186/1471-2458-13-931 PMC3851477

[19] David RJ, Collins JW Jr (1997) Differing birth weight among infants of U.S.-born blacks, African-born blacks, and U.S.-born whites. N Engl J Med 337(17):1209–1214. 10.1056/NEJM199710233371706 9337381 10.1056/NEJM199710233371706

[20] Shirley MK, Arthurs OJ, Seunarine KK et al (2022) Implications of leg length for metabolic health and fitness. Evol Med Public Health 10(1):316–324. 10.1093/emph/eoac023 35903461 10.1093/emph/eoac023 PMC9326181

[21] Shirley MK, Cole TJ, Arthurs OJ, Clark CA, Wells JCK (2020) Developmental origins of variability in pelvic dimensions: evidence from nulliparous South Asian women in the United Kingdom. Am J Hum Biol 32(2):e23340. 10.1002/ajhb.23340 31755611 10.1002/ajhb.23340 PMC7154657

[22] Wells JCK, Desoye G, Leon DA (2024) Reconsidering the developmental origins of adult disease paradigm: the “metabolic coordination of childbirth” hypothesis. Evol Med Public Health 12(1):50–66. 10.1093/emph/eoae002 38380130 10.1093/emph/eoae002 PMC10878253

[23] Zheng M, Hesketh KD, Vuillermin P et al (2022) Determinants of rapid infant weight gain: a pooled analysis of seven cohorts. Pediatr Obes 17(10):e12928. 10.1111/ijpo.12928 35510714 10.1111/ijpo.12928 PMC9540679

[24] Ahmed ML, Ong KK, Dunger DB (2009) Childhood obesity and the timing of puberty. Trends Endocrinol Metab 20(5):237–242. 10.1016/j.tem.2009.02.004 19541497 10.1016/j.tem.2009.02.004

[25] Nightingale CM, Rudnicka AR, Owen CG et al (2013) Influence of adiposity on insulin resistance and glycemia markers among U.K. Children of South Asian, black African-Caribbean, and white European origin: child heart and health study in England. Diabetes Care 36(6):1712–1719. 10.2337/dc12-1726 23315600 10.2337/dc12-1726 PMC3661837

[26] DeFronzo RA, Tripathy D (2009) Skeletal muscle insulin resistance is the primary defect in type 2 diabetes. Diabetes Care 32(Suppl 2):S157–S163. 10.2337/dc09-S302 19875544 10.2337/dc09-S302 PMC2811436

[27] Barr JG, Veena SR, Kiran KN et al (2010) The relationship of birthweight, muscle size at birth and post-natal growth to grip strength in 9-year-old Indian children: findings from the Mysore Parthenon study. J Dev Orig Health Dis 1(5):329–337. 10.1017/S2040174410000309 23750316 10.1017/S2040174410000309 PMC3672832

[28] Sun C, Kovacs P, Guiu-Jurado E (2021) Genetics of body fat distribution: comparative analyses in populations with European, Asian and African ancestries. Genes (Basel) 12(6):841. 10.3390/genes12060841 34072523 10.3390/genes12060841 PMC8228180

[29] Eastwood SV, Hemani G, Watkins SH, Scally A, Davey Smith G, Chaturvedi N (2024) Ancestry, ethnicity, and race: explaining inequalities in cardiometabolic disease. Trends Mol Med 30(6):541–551. 10.1016/j.molmed.2024.04.002 38677980 10.1016/j.molmed.2024.04.002

[30] Pomeroy E, Mushrif-Tripathy V, Cole TJ, Wells JCK, Stock JT (2019) Ancient origins of low lean mass among South Asians and implications for modern type 2 diabetes susceptibility. Sci Rep 9(1):10515. 10.1038/s41598-019-46960-9 31324875 10.1038/s41598-019-46960-9 PMC6642207

[31] Herle M, Micali N, Abdulkadir M et al (2020) Identifying typical trajectories in longitudinal data: modelling strategies and interpretations. Eur J Epidemiol 35(3):205–222. 10.1007/s10654-020-00615-6 32140937 10.1007/s10654-020-00615-6 PMC7154024

[32] Nightingale CM, Rudnicka AR, Owen CG et al (2013) Are ethnic and gender specific equations needed to derive fat free mass from bioelectrical impedance in children of South asian, black african-Caribbean and white European origin? Results of the assessment of body composition in children study. PLoS One 8(10):e76426. 10.1371/journal.pone.0076426 24204625 10.1371/journal.pone.0076426 PMC3799736

[33] Farmaki AE, Garfield V, Eastwood SV et al (2022) Type 2 diabetes risks and determinants in second-generation migrants and mixed ethnicity people of South Asian and African Caribbean descent in the UK. Diabetologia 65(1):113–127. 10.1007/s00125-021-05580-7 34668055 10.1007/s00125-021-05580-7 PMC8660755

