# Supplementary material for: Diabetogenic elevated childhood total fat in South Asian and Black African/Caribbean people relates to adverse early life growth and low socioeconomic position compared with White people in the UK
Source: Diabetologia. 2025 Jun 27;68(9):1958–68. doi: 10.1007/s00125-025-06473-9 (PMC12361325; doi:10.1007/s00125-025-06473-9)
Supplement: Supplementary file 1 — ESM Tables (PDF 269 KB ) [file 125_2025_6473_MOESM1_ESM.pdf]

ESM Table 1. Numbers for missing data by variable, subsequently imputed

	Variable	Not Missing	Missing
**Birth:**	Weight	13,980	13
	Maternal Height	13,814	179
	Maternal Weight	12,965	1,028
	Maternal Smoking	9,356	4,637
	Maternal Breastfeeding	9,336	4,657
	Area Deprivation	13,992	1
	Highest Parental Income	13,964	29
	Highest Parental Education	13,961	32
**Age 3:**	Height	11,633	2,360
	Weight	11,781	2,212
**Age 5:**	Height	13,502	491
	Weight	13,522	471
	Waist	13,420	573
**Age 7:**	Height	11,832	2,161
	Weight	11,803	2,190
	Waist	11,722	2,271
	Fat Mass	11,632	2,361
**Age 11:**	Height	11,087	2,906
	Weight	10,909	3,084
	Fat Mass	10,785	3,208
**Age 14:**	Height	9,604	4,389
	Weight	9,347	4,646
	Fat Mass	9,225	4,768
**Age 17:**	Height	8,205	5,788
	Weight	7,996	5,997
	Fat Mass	7,875	6,118

ESM Table 2a) Height, weight, fat mass index, and fat-free mass index (mean +/- standard deviation) by age, ethnicity, and sex

	Age	Boys						Girls					
		White	Indian	Pakistani	Bangladeshi	Black Caribbean	Black African	White	Indian	Pakistani	Bangladeshi	Black Caribbean	Black African
Height (cm)	3	95.93 ± 4.65	96.44 ± 4.84	96.62 ± 4.49	96.27 ± 6.51	96.89 ± 5.26	98.74 ± 5.77	94.74 ± 4.55	95.57 ± 6.12	95.54 ± 5.14	95.68 ± 4.75	96.94 ± 4.33	98.80 ± 5.76
	5	111.08 ± 5.46	111.98 ± 6.08	111.88 ± 5.67	111.67 ± 6.75	112.52 ± 6.79	114.48 ± 6.96	110.18 ± 5.51	111.21 ± 6.89	110.29 ± 5.62	110.09 ± 6.23	112.79 ± 4.85	114.35 ± 6.06
	7	124.07 ± 6.24	125.21 ± 6.75	124.60 ± 6.36	123.95 ± 8.36	125.82 ± 7.17	127.85 ± 7.96	123.17 ± 6.30	124.05 ± 7.62	123.08 ± 6.12	122.42 ± 6.74	125.96 ± 4.77	128.03 ± 7.44
	11	145.98 ± 8.11	147.54 ± 9.46	146.64 ± 8.07	145.51 ± 8.97	147.86 ± 9.31	149.68 ± 9.83	146.83 ± 8.58	147.79 ± 10.43	146.21 ± 8.39	145.76 ± 8.99	151.04 ± 7.47	151.95 ± 9.71
	14	167.12 ± 9.66	167.24 ± 9.30	166.45 ± 8.80	165.79 ± 10.50	167.49 ± 10.65	169.24 ± 10.75	161.82 ± 8.20	160.20 ± 9.50	159.00 ± 7.39	156.98 ± 8.60	162.78 ± 7.59	163.56 ± 9.07
	17	177.04 ± 8.39	175.27 ± 8.17	174.80 ± 7.03	172.21 ± 9.96	175.40 ± 9.25	176.77 ± 9.51	164.36 ± 8.66	161.88 ± 9.41	160.92 ± 6.78	158.41 ± 8.20	163.87 ± 7.36	164.48 ± 8.21
Weight (Kg)	0	3.46 ± 0.68	3.03 ± 0.89	3.15 ± 0.65	3.13 ± 0.61	3.27 ± 0.87	3.37 ± 0.73	3.34 ± 0.66	2.96 ± 0.81	3.08 ± 0.67	2.97 ± 0.95	3.15 ± 0.72	3.16 ± 0.72
	3	15.30 ± 2.26	14.70 ± 2.84	14.89 ± 2.56	15.02 ± 2.92	16.11 ± 2.90	16.33 ± 3.00	14.63 ± 2.27	14.15 ± 2.98	14.54 ± 2.62	14.66 ± 3.49	15.64 ± 2.92	16.45 ± 3.70
	5	20.23 ± 3.12	19.88 ± 4.24	19.81 ± 4.05	20.21 ± 4.51	21.87 ± 4.46	21.85 ± 4.30	19.80 ± 3.31	19.27 ± 3.68	19.46 ± 3.73	19.70 ± 4.80	21.38 ± 3.73	22.63 ± 4.65
	7	25.50 ± 4.91	25.70 ± 6.68	25.43 ± 6.80	25.40 ± 6.48	28.05 ± 7.00	28.35 ± 6.78	25.33 ± 5.33	24.97 ± 5.64	24.72 ± 5.54	25.00 ± 6.83	27.85 ± 5.67	29.99 ± 7.95
	11	40.51 ± 10.66	42.44 ± 13.69	42.20 ± 12.59	40.97 ± 12.63	45.71 ± 14.97	45.06 ± 13.12	42.07 ± 11.62	41.07 ± 12.15	42.11 ± 12.27	42.06 ± 12.67	47.89 ± 12.91	50.64 ± 14.80
	14	58.57 ± 15.12	59.48 ± 18.33	59.03 ± 16.40	58.10 ± 15.59	64.39 ± 20.50	61.69 ± 16.73	57.81 ± 14.93	53.57 ± 13.64	56.23 ± 16.39	54.72 ± 15.45	62.67 ± 17.58	65.42 ± 18.54
	17	71.87 ± 19.48	71.97 ± 21.59	71.84 ± 20.83	68.83 ± 20.02	75.79 ± 21.55	73.81 ± 19.86	64.02 ± 17.20	58.39 ± 17.12	62.01 ± 18.93	59.39 ± 18.34	67.54 ± 20.39	70.75 ± 20.62
FMI (kg/m^2)	7	3.34 ± 1.38	3.58 ± 1.89	3.63 ± 2.13	3.59 ± 1.62	4.02 ± 2.36	3.86 ± 1.47	3.74 ± 1.66	3.75 ± 1.65	3.81 ± 1.80	4.00 ± 2.17	4.33 ± 1.94	4.99 ± 2.50
	11	3.91 ± 2.52	4.83 ± 3.23	4.89 ± 3.09	4.55 ± 3.16	5.20 ± 4.63	4.91 ± 3.06	4.96 ± 2.92	4.75 ± 2.75	5.38 ± 3.08	5.50 ± 3.25	6.00 ± 3.54	6.87 ± 3.69
	14	3.66 ± 2.76	4.50 ± 3.74	4.55 ± 3.38	4.17 ± 2.95	4.91 ± 4.49	4.22 ± 2.97	6.24 ± 3.52	5.62 ± 3.00	6.65 ± 4.02	6.69 ± 3.85	7.48 ± 4.35	8.07 ± 4.38
	17	3.94 ± 3.53	4.68 ± 4.44	4.95 ± 4.30	4.41 ± 3.67	5.15 ± 4.76	4.63 ± 3.55	7.05 ± 4.43	6.23 ± 3.92	7.56 ± 5.02	7.10 ± 4.80	8.14 ± 5.73	9.01 ± 5.13
FFMI (kg/m^2)	7	13.16 ± 1.16	12.69 ± 1.57	12.63 ± 1.51	12.83 ± 1.65	13.56 ± 1.45	13.34 ± 1.46	12.87 ± 1.15	12.39 ± 1.32	12.42 ± 1.24	12.56 ± 1.50	13.14 ± 1.13	13.20 ± 1.41
	11	14.97 ± 1.78	14.47 ± 2.08	14.54 ± 1.88	14.63 ± 2.13	15.53 ± 1.95	15.01 ± 2.03	14.41 ± 1.62	13.91 ± 1.96	14.14 ± 1.88	14.16 ± 1.93	14.86 ± 1.62	14.91 ± 1.70
	14	17.21 ± 2.39	16.64 ± 2.47	16.66 ± 2.50	16.88 ± 2.57	17.88 ± 2.74	17.20 ± 2.63	15.78 ± 1.97	15.21 ± 2.20	15.50 ± 2.12	15.47 ± 2.26	16.08 ± 2.04	16.28 ± 1.85
	17	18.96 ± 2.83	18.69 ± 2.49	18.51 ± 2.81	18.73 ± 2.73	19.43 ± 2.80	18.94 ± 3.40	16.63 ± 2.32	15.98 ± 2.48	16.33 ± 2.34	16.55 ± 2.88	16.96 ± 2.51	17.04 ± 2.32

ESM Table 2b) Standardised height, weight, fat mass index, and fat-free mass index by age ethnicity, and sex

	Age	Boys						Girls					
		White	Indian	Pakistani	Bangladeshi	Black Caribbean	Black African	White	Indian	Pakistani	Bangladeshi	Black Caribbean	Black African
Height (cm)	3	-	0.11	0.14	0.07	0.20	0.59	-	0.18	0.18	0.21	0.48	0.89
	5	-	0.16	0.14	0.10	0.26	0.61	-	0.19	0.02	-0.02	0.47	0.76
	7	-	0.18	0.08	-0.02	0.27	0.59	-	0.14	-0.01	-0.12	0.44	0.77
	11	-	0.19	0.08	-0.06	0.23	0.45	-	0.11	-0.07	-0.12	0.49	0.60
	14	-	0.01	-0.07	-0.13	0.04	0.22	-	-0.20	-0.35	-0.59	0.12	0.21
	17	-	-0.21	-0.26	-0.56	-0.19	-0.03	-	-0.29	-0.40	-0.69	-0.06	0.01
Weight (Kg)	0	-	-0.62	-0.45	-0.47	-0.27	-0.13	-	-0.58	-0.40	-0.56	-0.29	-0.27
	3	-	-0.26	-0.18	-0.12	0.35	0.45	-	-0.21	-0.04	0.01	0.44	0.80
	5	-	-0.11	-0.13	-0.01	0.51	0.51	-	-0.16	-0.10	-0.03	0.48	0.86
	7	-	0.04	-0.01	-0.02	0.51	0.57	-	-0.07	-0.11	-0.06	0.47	0.87
	11	-	0.18	0.16	0.04	0.48	0.42	-	-0.09	0.00	0.00	0.50	0.74
	14	-	0.06	0.03	-0.03	0.38	0.20	-	-0.28	-0.11	-0.21	0.33	0.51
	17	-	0.00	0.00	-0.15	0.20	0.10	-	-0.33	-0.12	-0.27	0.20	0.39
FMI (kg/m^2)	7	-	0.17	0.21	0.18	0.48	0.37	-	0.01	0.04	0.16	0.35	0.75
	11	-	0.36	0.38	0.25	0.50	0.39	-	-0.07	0.14	0.18	0.36	0.65
	14	-	0.30	0.32	0.18	0.44	0.20	-	-0.18	0.12	0.13	0.35	0.52
	17	-	0.20	0.28	0.13	0.34	0.19	-	-0.19	0.11	0.01	0.25	0.44
FFMI (kg/m^2)	7	-	-0.40	-0.44	-0.28	0.34	0.15	-	-0.42	-0.39	-0.27	0.23	0.28
	11	-	-0.27	-0.24	-0.19	0.30	0.02	-	-0.31	-0.17	-0.15	0.28	0.31
	14	-	-0.23	-0.23	-0.14	0.27	-0.01	-	-0.29	-0.14	-0.16	0.15	0.25
	17	-	-0.09	-0.15	-0.08	0.16	-0.01	-	-0.28	-0.13	-0.03	0.14	0.18

ESM Table 3a. Accounting for ethnic differences (using white group as comparator) in FMI at age 7 in boys, coefficient and 95% CI, p value

	**Indian**		**Pakistani**		**Bangladeshi**		**Black Caribbean**		**Black African**	
	Coefficient (95% CI)	*P value*	*Coefficient (95% CI)*	*P value*	*Coefficient (95% CI)*	*P value*	*Coefficient (95% CI)*	*P value*	*Coefficient (95% CI)*	*P value*
**Model 1**										
Ethnicity	0.24 (-0.04, 0.53)	0.097	0.29 (0.06, 0.53)	0.014	0.25 (-0.02, 0.52)	0.071	0.68 (0.16, 1.21)	0.01	0.52 (0.27, 0.78)	<0.001
**Model 2**										
Ethnicity	0.14 (-0.14, 0.43)	0.322	0.24 (0.01, 0.47)	0.042	0.19 (-0.08, 0.46)	0.175	0.58 (0.07, 1.08)	0.026	0.45 (0.20, 0.70)	<0.001
Weight gain to age 3	0.17 (0.13, 0.22)	<0.001	0.18 (0.13, 0.22)	<0.001	0.17 (0.12, 0.22)	<0.001	0.17 (0.13, 0.22)	<0.001	0.17 (0.13, 0.22)	<0.001
**Model 3**										
Ethnicity	0.34 (0.08, 0.60)	0.012	0.2 7(0.05, 0.49)	0.017	0.17 (-0.09, 0.43)	0.194	0.45 (-0.02, 0.93)	0.062	0.31 (0.08, 0.54)	0.009
Weight gain to age 3	0.48 (0.39, 0.58)	<0.001	0.49 (0.40, 0.59)	<0.001	0.48 (0.39, 0.58)	<0.001	0.48 (0.39, 0.58)	<0.001	0.49 (0.39, 0.58)	<0.001
Maternal height	-2.54 (-3.07, -2.02)	<0.001	-2.58 (-3.10, -2.06)	<0.001	-2.61 (-3.13, -2.09)	<0.001	-2.62 (-3.14, -2.09)	<0.001	-2.56 (-3.08, -2.04)	<0.001
Maternal weight	0.02 (0.02, 0.02)	<0.001	0.0 2(0.02, 0.02)	<0.001	0.02 (0.02, 0.02)	<0.001	0.02 (0.02, 0.02)	<0.001	0.02 (0.02, 0.02)	<0.001
Breast fed to 4 months	-0.09 (-0.18, 0.00)	0.061	-0.08 (-0.18, 0.01)	0.069	-0.08 (-0.18, 0.01)	0.086	-0.08 (-0.17, 0.02)	0.114	-0.08 (-0.17, 0.02)	0.101
Maternal smoking	0.10 (-0.03, 0.23)	0.134	0.10 (-0.03, 0.23)	0.126	0.10 (-0.03, 0.23)	0.12	0.11 (-0.02, 0.24)	0.108	0.10 (-0.03, 0.23)	0.124
Birthweight	0.85 (0.73, 0.97)	<0.001	0.87 (0.74, 0.99)	<0.001	0.85 (0.73, 0.97)	<0.001	0.85 (0.73, 0.97)	<0.001	0.85 (0.73, 0.97)	<0.001
Area deprivation	-0.02 (-0.03, 0.00)	0.022	-0.01 (-0.03, 0.00)	0.058	-0.01 (-0.03, 0.00)	0.031	-0.01 (-0.03, 0.00)	0.032	-0.02 (-0.03, 0.00)	0.018
Income	-0.05 (-0.08, -0.02)	<0.001	-0.05 (-0.08, -0.02)	<0.001	-0.05 (-0.08, -0.02)	<0.001	-0.05 (-0.08, -0.02)	<0.001	-0.05 (-0.07, -0.02)	0.001

ESM Table 3b. Accounting for ethnic differences (using white group as comparator) in FMI at age 7 in girls, coefficient and 95% CI, p value

	**Indian**		**Pakistani**		**Bangladeshi**		**Black Caribbean**		**Black African**	
	Coefficient (95% CI)	P value	Coefficient (95% CI)	P value	Coefficient (95% CI)	P value	Coefficient (95% CI)	P value	Coefficient (95% CI)	P value
**Model 1**										
Ethnicity	0.01 (-0.24, 0.26)	0.942	0.07 (-0.13, 0.27)	0.503	0.26 (-0.11, 0.62)	0.164	0.59 (0.16, 1.01)	0.008	1.24 (0.81, 1.68)	<0.001
**Model 2**										
Ethnicity	-0.09 (-0.36, 0.18)	0.516	-0.02 (-0.22, 0.18)	0.851	0.10 (-0.27, 0.46)	0.601	0.40 (-0.02, 0.82)	0.059	1.06 (0.65, 1.48)	<0.001
Weight gain to age 3	0.20 (0.15, 0.26)	<0.001	0.21 (0.15, 0.26)	<0.001	0.21 (0.15, 0.27)	<0.001	0.21 (0.15, 0.26)	<0.001	0.21 (0.16, 0.27)	<0.001
**Model 3**										
Ethnicity	0.20 (-0.08, 0.47)	0.158	-0.02 (-0.20, 0.17)	0.847	0.02 (-0.32, 0.37)	0.901	0.09 (-0.34, 0.52)	0.683	0.72 (0.33, 1.11)	<0.001
Weight gain to age 3	0.61 (0.48, 0.73)	<0.001	0.60 (0.47, 0.72)	<0.001	0.61 (0.49, 0.74)	<0.001	0.59 (0.47, 0.71)	<0.001	0.62 (0.49, 0.75)	<0.001
Maternal height	-2.26 (-2.91, -1.60)	<0.001	-2.21 (-2.86, -1.56)	<0.001	-2.24 (-2.90, -1.57)	<0.001	-2.19 (-2.85, -1.53)	<0.001	-2.14 (-2.80, -1.49)	<0.001
Maternal weight	0.02 (0.02, 0.02)	<0.001	0.02 (0.02, 0.03)	<0.001	0.02 (0.02, 0.02)	<0.001	0.02 (0.02, 0.03)	<0.001	0.02 (0.02, 0.02)	<0.001
Breast fed to 4 months	-0.12 (-0.21, -0.03)	0.012	-0.11 (-0.21, -0.02)	0.018	-0.11(-0.20, -0.01)	0.025	-0.11 (-0.21, -0.02)	0.023	-0.11 (-0.21, -0.02)	0.021
Maternal smoking	0.17 (0.03, 0.30)	0.017	0.17 (0.04, 0.31)	0.012	0.18 (0.04, 0.31)	0.01	0.17 (0.04, 0.31)	0.013	0.18 (0.04, 0.31)	0.01
Birthweight	1.19 (1.04, 1.35)	<0.001	1.17 (1.01, 1.33)	<0.001	1.20 (1.04, 1.36)	<0.001	1.16 (1.01, 1.32)	<0.001	1.20 (1.04, 1.36)	<0.001
Area deprivation	-0.02 (-0.03, -0.01)	0.008	-0.02 (-0.04, -0.01)	0.004	-0.02 (-0.04, -0.01)	0.005	-0.02 (-0.04, -0.01)	0.006	-0.02 (-0.03, 0.00)	0.011
Income	-0.08 (-0.11, -0.04)	<0.001	-0.07 (-0.10, -0.04)	<0.001	-0.08 (-0.11, -0.04)	<0.001	-0.07 (-0.11, -0.04)	<0.001	-0.08 (-0.11, -0.04)	<0.001

ESM Table 4a. Accounting for ethnic differences (using white group as comparator) in FFMI at age 7 in boys, coefficient and 95% CI, p value

	Indian		Pakistani		Bangladeshi		Black Caribbean		Black African	
	Coefficient (95% CI)	P value	Coefficient (95% CI)	P value	Coefficient (95% CI)	P value	Coefficient (95% CI)	P value	Coefficient (95% CI)	P value
**Model 1**										
Ethnicity	-0.47 (-0.71, -0.23)	<0.001	-0.53 (-0.70, -0.36)	<0.001	-0.33 (-0.60, -0.05)	0.019	0.41 (0.09, 0.73)	0.013	0.18 (-0.07, 0.44)	0.159
**Model 2**										
Ethnicity	-0.51 (-0.75, -0.28)	<0.001	-0.55 (-0.72, -0.38)	<0.001	-0.36 (-0.63, -0.08)	0.011	0.36 (0.04, 0.68)	0.03	0.15 (-0.10, 0.40)	0.247
Weight gain to age 3	0.08 (0.04, 0.12)	<0.001	0.08 (0.04, 0.12)	<0.001	0.08 (0.04, 0.12)	<0.001	0.08 (0.04, 0.12)	<0.001	0.08 (0.04, 0.12)	<0.001
**Model 3**										
Ethnicity	-0.30 (-0.51, -0.08)	0.007	-0.44 (-0.60, -0.29)	<0.001	-0.28 (-0.56, 0.00)	0.054	0.25 (-0.03, 0.54)	0.084	0.03 (-0.19, 0.26)	0.788
Weight gain to age 3	0.42 (0.32, 0.51)	<0.001	0.42 (0.33, 0.52)	<0.001	0.42 (0.32, 0.51)	<0.001	0.41 (0.32, 0.51)	<0.001	0.42 (0.32, 0.51)	<0.001
Maternal height	-1.73 (-2.18, -1.28)	<0.001	-1.74 (-2.19, -1.29)	<0.001	-1.75 (-2.20, -1.30)	<0.001	-1.75 (-2.20, -1.30)	<0.001	-1.73 (-2.18, -1.28)	<0.001
Maternal weight	0.01 (0.01, 0.02)	<0.001	0.01 (0.01, 0.02)	<0.001	0.01 (0.01, 0.02)	<0.001	0.01 (0.01, 0.02)	<0.001	0.01 (0.01, 0.02)	<0.001
Breast fed to 4 months	0.00 (-0.07, 0.07)	0.971	0.00 (-0.07, 0.08)	0.944	0.00 (-0.07, 0.08)	0.92	0.01 (-0.07, 0.08)	0.873	0.00 (-0.07, 0.08)	0.942
Maternal smoking	0.08 (-0.02, 0.19)	0.104	0.09 (-0.01, 0.20)	0.083	0.08 (-0.02, 0.19)	0.108	0.09 (-0.02, 0.19)	0.098	0.08 (-0.02, 0.19)	0.106
Birthweight	0.91 (0.80, 1.02)	<0.001	0.91 (0.80, 1.02)	<0.001	0.90 (0.79, 1.01)	<0.001	0.91 (0.79, 1.02)	<0.001	0.91 (0.80, 1.02)	<0.001
Area deprivation	0.00 (-0.02, 0.01)	0.364	0.00 (-0.01, 0.01)	0.422	0.00 (-0.01, 0.01)	0.401	0.00 (-0.01, 0.01)	0.436	0.00 (-0.01, 0.01)	0.408
Income	-0.04 (-0.06, -0.01)	0.002	-0.04 (-0.06, -0.01)	0.003	-0.04 (-0.06, -0.01)	0.003	-0.04 (-0.06, -0.01)	0.003	-0.03 (-0.06, -0.01)	0.005

ESM Table 4b. Accounting for ethnic differences (using white group as comparator) in FFMI at age 7 in girls, coefficient and 95% CI, p value

	**Indian**		**Pakistani**		**Bangladeshi**		**Black Caribbean**		**Black African**	
	Coefficient (95% CI)	P value	Coefficient (95% CI)	P value	Coefficient (95% CI)	P value	Coefficient (95% CI)	P value	Coefficient (95% CI)	P value
**Model 1**										
Ethnicity	-0.48 (-0.68, -0.28)	<0.001	-0.45 (-0.59, -0.31)	<0.001	-0.31 (-0.56, -0.06)	0.014	0.26 (0.01, 0.52)	0.039	0.33 (0.08, 0.57)	0.009
**Model 2**										
Ethnicity	-0.52 (-0.72, -0.31)	<0.001	-0.49 (-0.62, -0.35)	<0.001	-0.38 (-0.63, -0.12)	0.004	0.19 (-0.06, 0.44)	0.129	0.26 (0.02, 0.49)	0.036
Weight gain to age 3	0.08 (0.04, 0.11)	<0.001	0.08 (0.05, 0.12)	<0.001	0.08 (0.05, 0.12)	<0.001	0.08 (0.05, 0.12)	<0.001	0.08 (0.05, 0.12)	<0.001
**Model 3**										
Ethnicity	-0.32 (-0.54, -0.11)	0.004	-0.44 (-0.57, -0.31)	<0.001	-0.39 (-0.64, -0.14)	0.002	0.00 (-0.25, 0.25)	0.99	0.05 (-0.17, 0.28)	0.641
Weight gain to age 3	0.39 (0.30, 0.47)	<0.001	0.38 (0.30, 0.47)	<0.001	0.40 (0.31, 0.48)	<0.001	0.38 (0.30, 0.46)	<0.001	0.40 (0.31, 0.49)	<0.001
Maternal height	-1.60 (-2.08, -1.13)	<0.001	-1.56 (-2.03, -1.09)	<0.001	-1.58 (-2.05, -1.10)	<0.001	-1.55 (-2.03, -1.08)	<0.001	-1.56 (-2.03, -1.09)	<0.001
Maternal weight	0.01 (0.01, 0.01)	<0.001	0.01 (0.01, 0.01)	<0.001	0.01 (0.01, 0.01)	<0.001	0.01 (0.01, 0.01)	<0.001	0.01 (0.01, 0.01)	<0.001
Breast fed to 4 months	-0.02 (-0.09, 0.06)	0.633	-0.01 (-0.08, 0.06)	0.796	-0.01 (-0.08, 0.07)	0.829	-0.01 (-0.09, 0.07)	0.786	-0.01 (-0.09, 0.06)	0.741
Maternal smoking	0.10 (0.01, 0.19)	0.028	0.11 (0.02, 0.20)	0.017	0.11 (0.02, 0.20)	0.015	0.11 (0.02, 0.19)	0.018	0.11 (0.02, 0.20)	0.016
Birthweight	0.92 (0.81, 1.02)	<0.001	0.90 (0.79, 1.01)	<0.001	0.92 (0.82, 1.03)	<0.001	0.90 (0.79, 1.00)	<0.001	0.92 (0.82, 1.03)	<0.001
Area deprivation	-0.01 (-0.02, 0.00)	0.088	-0.01 (-0.02, 0.00)	0.06	-0.01 (-0.02, 0.00)	0.069	-0.01 (-0.02, 0.00)	0.08	-0.01 (-0.02, 0.00)	0.09
Income	-0.03 (-0.06, -0.01)	0.004	-0.03 (-0.05, -0.01)	0.008	-0.03 (-0.05, -0.01)	0.005	-0.03 (-0.05, -0.01)	0.005	-0.03 (-0.05, -0.01)	0.007
